# Risk of major depressive disorder in Japanese cancer patients: A matched cohort study using employer‐based health insurance claims data

**DOI:** 10.1002/pon.5509

**Published:** 2020-09-01

**Authors:** Tatsuo Akechi, Izumi Mishiro, Shinji Fujimoto, Katsuhito Murase

**Affiliations:** ^1^ Department of Psychiatry and Cognitive‐Behavioral Medicine Nagoya City University Graduate School of Medical Sciences Nagoya Japan; ^2^ Japan Medical Office Takeda Pharmaceutical Company Limited Tokyo Japan

**Keywords:** administrative claims healthcare, cancer, depressive disorder major, epidemiology, neoplasms, oncology, psycho‐oncology

## Abstract

**Objective:**

Patients with cancer are at high risk of depression. However, the risk of major depressive disorder (MDD) after cancer diagnosis has not been studied in a population setting in Japan. This cohort study used a Japanese medical claims database to examine time to MDD in cancer patients and the risk of MDD (hazard ratio; HR) compared with matched cancer‐free controls.

**Methods:**

Primary endpoint was time to MDD (starting 6 months before cancer diagnosis) in adult (18–74 years) cancer patients; secondary endpoint was time to MDD (6 months before to 12 months after cancer diagnosis) in a matched cohort of cancer patients and cancer‐free controls. Multivariate analyses were performed to determine HRs for all cancers and for each cancer site.

**Results:**

Of 35 008 cancer patients (mean age, 53.3 years), 2201 (6.3%) were diagnosed with MDD within 66 months. Matched cancer patients (n = 30 372) had an elevated risk of MDD compared with cancer‐free controls (n = 303 720; HR [95% confidence interval] 2.96 [2.77–3.16]). MDD risk was highest in patients with multiple cancers, pancreatic cancer, and brain cancer. Compared with middle‐aged patients, risk was higher in patients <40 years old and lower in patients ≥65 years old; risk tended to be higher in women than in men.

**Conclusions:**

Compared with cancer‐free individuals, Japanese patients with cancer, mostly <65 years old, had an almost threefold higher risk of developing MDD within 12 months of cancer diagnosis. Physicians should watch for MDD in cancer patients and treat when necessary.

## 1 BACKGROUND

Being diagnosed with cancer is a stressful event that often leads to anxiety and depression.^1^ The prevalence of depression in cancer patients, as reported in systematic reviews and meta‐analyses, ranges from as low as 1% to as high as 77.5% and depends on the clinical setting, the type and severity of cancer, the method used to diagnose depression, and the expertise level of the diagnosing physician.^2^, ^3^, ^4^ A meta‐analysis of 70 studies involving 10 071 individuals with cancer in oncological and hematological settings reported that the prevalence of major depression as defined by Diagnostic and Statistical Manual of Mental Disorders criteria (assessed in 52 of the studies) was 14.9% (95% confidence interval [CI] 12.2‐17.7).^3^ Importantly, depression in patients with cancer is associated with an elevated risk of cancer mortality^5^ and an up to 24‐fold greater risk of suicide or death by external injury, particularly in the first week after cancer diagnosis.^6^, ^7^ Depression also reduces adherence to cancer treatments,^8^ is a psychological burden on the family,^9^ and increases healthcare utilization, including hospitalizations.^10^

Although the prevalence of depression in cancer patients has been well documented, only one large, well‐controlled, matched cohort study, conducted in Sweden, has examined the incidence of newly developed mental disorders following a cancer diagnosis. In that study, an increased risk of mental disorders, including depression, began up to 10 months before cancer diagnosis, peaked immediately after diagnosis, and remained elevated above baseline for up to 10 years.^11^ Further, the risk of mental disorders during the first year after cancer diagnosis was greatest among patients aged 40–64 years (ie, middle‐aged patients).^11^

One in two people in Japan are estimated to be diagnosed with cancer in their lifetimes.^12^ Although its effect on mortality has decreased in recent years, cancer has been the leading cause of death in Japan since 1981.^13^ In a Japanese retrospective study, almost half of patients with eight common types of cancer were prescribed psychotropic drugs, including antidepressants and benzodiazepines, soon after their cancer diagnoses.^14^ These results suggest that Japanese patients with cancer are at high risk of developing depression. However, population‐based data on the incidence of depression in Japan are limited to the World Mental Health Survey,^15^, ^16^ and no data exist on the incidence of depression in cancer patients. Furthermore, because depression is less prevalent in Japan than in other countries,^15^ possibly because of social stigma associated with mental disorders,^17^ it is important to determine the rate of depression among Japanese patients with cancer.

To expand our understanding of the relationship between cancer and depression, particularly in Japan, we used a large, nationwide, employer‐based health insurance database to estimate the time to clinically diagnosed major depressive disorder (MDD) in more than 30 000 Japanese patients with any type of newly diagnosed cancer. In addition, we used a matched cohort of cancer patients and cancer‐free controls to determine hazard ratios (HRs) for the development of MDD for all cancers and for individual cancer types. To our knowledge, this is the first study to determine HRs for depression across a broad range of cancer types.

## 2 MATERIALS AND METHODS

### 2.1 Study design

This cohort study used a commercially available, anonymized database of workers' medical services and prescriptions collected from multiple health insurance societies in Japan (JMDC database; JMDC Inc., Tokyo, Japan).^18^ The JMDC database provides data on patient demographics, disease diagnoses based on International Statistical Classification of Diseases and Related Health Problems, 10th revision (ICD‐10) codes, prescriptions, and medical procedures. In Japan, every person is covered by either a national or an employer‐based comprehensive health insurance scheme.^19^ The JMDC database covers employees of medium to large companies and their dependents (family members living in the same household who earn <1.3 million yen per year and who are <75 years old). In Japan, public health insurance systems exist for the elderly (≥75 years old) and some specific professions (eg, farmers, public officers).^20^ Therefore, these individuals are not included in the employer‐based JMDC database. The study analyzed data between January 2011 and September 2018 for individuals who met the eligibility criteria described below. Because this study used a database in which only anonymized information was available to the investigators, and in accordance with the Ethical Guidelines for Medical and Health Research Involving Human Subjects in Japan, institutional ethics approval and informed consent were not required.

### 2.2 Study population

#### 2.2.1 Adult cancer patient cohort

Time to depression was investigated in the adult cancer patient cohort. Adult (aged 18‐74 years) cancer patients had a new diagnosis of cancer during the enrollment period (January 2012‐September 2017); no documented, definitive diagnosis of cancer in the 12 months before the index month (month/year of cancer diagnosis); ≥2 diagnoses (ie, ≥2 health insurance claims issued for cancer diagnosis), including the initial diagnosis, for the same cancer site within 3 months of the index month; no diagnosis of MDD between 6 and 12 months before the index month; and continuous health insurance enrollment for ≥12 months before the index month to ensure access to complete records. Cancer diagnosis and site were identified by ICD‐10 codes (Table S1). The adult cancer patient cohort was followed up for as long as possible.

#### 2.2.2 Matched cohort of cancer patients and cancer‐free controls

The HR for development of depression over 1 year after cancer diagnosis in cancer patients compared with cancer‐free controls was investigated in the matched cohort. In this matched cohort, the cancer group included those adult cancer patients who had continuous health insurance enrollment for ≥12 months after the index month. The cancer‐free group included individuals with no cancer diagnosis who were matched to cancer patients according to age, sex, and working status (working vs non‐working in the health insurance scheme). Cancer‐free individuals also had to have continuous health insurance enrollment for ≥12 months before and ≥12 months after the index month of their matched cancer patient and no diagnosis of MDD between 6 and 12 months before the index month. A random sampling without replacement was performed at a ratio of cancer patients to non‐cancer controls of 1:10. This matching ratio was based on the number of patients targeted for matching (approximately 30,000 cancer patients) and the availability of well‐matched controls, and is in line with the ratio used in the Swedish cohort study.^11^

### 2.3 Outcome measures

The primary endpoint was the time from the start of observation to the onset of MDD, defined as the month with the first appearance of a documented diagnosis of MDD (ICD‐10 code F32 [“Depressive episode”] or F33 [“Major depressive disorder, recurrent”]), in the all‐adult cancer patient cohort. The observation period started 6 months before the index month and continued for as long as possible. Data were censored at the end of the observation period for patients without onset of MDD. A secondary endpoint of the study was the time from the start of observation to the onset of MDD within the 18‐month observation period (6 months before and 12 months after the index month) in the matched cohort. The observation periods started 6 months before the index month because cancer‐related MDD may develop as a result of patients' concerns with their health and uncertainty while undergoing diagnostic testing.

Data on demographic and other baseline characteristics of patients were also collected, including site of cancer occurrence (if multiple sites were diagnosed at baseline, then the site was classified as “multiple categories”), sex, age, insurance membership category, observable months, index month, and cancer therapy within 2 months of the index month (chemotherapy status, radiation therapy status, surgery with ≥5 days of hospitalization; used as an indicator of cancer severity).

### 2.4 Statistical analysis

Analysis of time to onset of MDD in all adult cancer patients (primary endpoint) and in the matched cohort cancer and cancer‐free groups (secondary endpoint) was performed using the Kaplan‐Meier method. The population at risk, cumulative number of events, cumulative number of censored patients, and percentage (95% CI) of patients without MDD (survival) are presented for every 6 months from the start of the observation period. The cumulative incidence of MDD was compared between the cancer group and the cancer‐free group using a log‐rank test with a two‐sided significance level of 5%. In the matched cohort, multivariate analysis was performed using a Cox proportional hazards model to estimate the HR for onset of MDD in the matched cancer group versus the cancer‐free group for all cancers and for each cancer site. The following variables were used for multivariate analysis: (a) cancer or cancer‐free group; (b) sex/age interaction term (sex/age were included in the covariates as interaction terms as the effects of age may differ depending on sex; if the site of cancer was breast, uterine cervix, uterine corpus, ovary, or other female genitalia, a separate estimate was performed using “female/40‐64 years of age” as the reference in the sex/age interaction term); and (c) working status (working vs non‐working). An additional multivariate subgroup analysis was conducted in the matched cohort cancer group only, with covariates of sex, age, membership category, chemotherapy status, radiotherapy status, and surgery requiring hospitalization for ≥5 days status. If the HR or CI of the HR was <0.01 or >999.99, it is reported as “not evaluable.” The data warehouse platform was Netezza N2002‐010 7.1.0.4.P2 (IBM, Armonk, New York), and statistical analysis was performed using SAS version 9.4 (SAS Institute, Cary, North Carolina).

## 3 RESULTS

### 3.1 Demographic and baseline clinical characteristics

**FIGURE 1:**
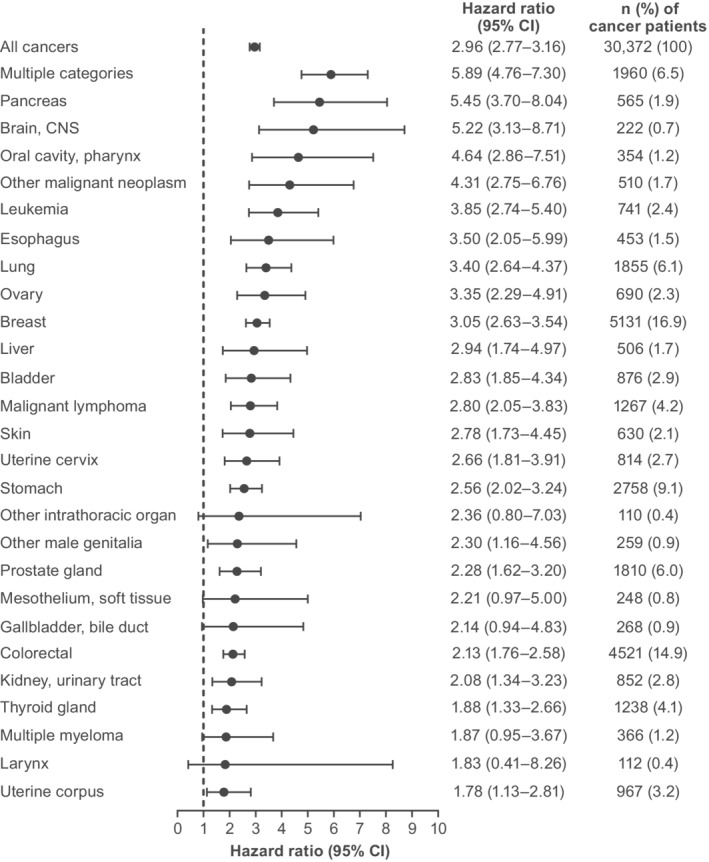
Hazard ratios and 95% CIs of the risk of major depressive disorder in the matched cohort (cancer group vs cancer‐free group) for each cancer type. Not shown are cancers of the small intestine (n = 103; 0.3%); bone and articular cartilage (n = 57; 0.2%); nasal cavity, paranasal sinus, and middle ear (n = 50; 0.2%); other endocrine gland (n = 38; 0.1%); other female genitalia (n = 25; 0.1%); other digestive organ (n = 10; <0.1%); and eye (n = 6; <0.1%). CI, confidence interval; CNS, central nervous system

There were 35 008 adult patients with newly diagnosed and confirmed cancer during the enrollment period and no recent history of MDD (Figure S1; Table 1), with a median (range) observable period of 32 (7‐87) months. Of these patients, 30 372 had continuous insurance enrollment during the 18‐month observation period and were included in the cancer group of the matched cohort (Figure S1; Table 1). A cancer‐free group of 303 720 individuals was derived after matching against the patients with cancer (Table 1). Most patients in the matched cohort (76.5%) were between 40 and 64 years of age and most (64.8%) were workers. There were slightly more men than women (51.2% vs 48.8%). Most of the cancer patients had not had chemotherapy (79.1%). Patients in the matched cohort cancer group had a broad range of cancer types, with the most common being breast (16.9% of all patients; 34.5% of female patients), colorectum (14.9%), stomach (9.1%), lung (6.1%), and prostate (6.0%; 11.6% of male patients); 6.5% of patients had cancers in multiple categories (Figure 1).

**TABLE 1:** Background and characteristics of patients with cancer and cancer‐free individuals in Japan

		Matched cohort	
Variables	All cancer patients n = 35 008	Cancer group n = 30 372	Cancer‐free group n = 303 720
Sex			
Male	18 297 (52.3)	15 545 (51.2)	155 450 (51.2)
Female	16 711 (47.7)	14 827 (48.8)	148 270 (48.8)
Age			
Mean (SD)	53.3 (10.8)	52.5 (10.6)	52.5 (10.6)
Median (range)	54.0 (18–74)	53.0 (18–74)	53.0 (18–74)
<40	3698 (10.6)	3415 (11.2)	34 150 (11.2)
40 to 64	26 151 (74.7)	23 221 (76.5)	232 210 (76.5)
≥65	5159 (14.7)	3736 (12.3)	37 360 (12.3)
Working status			
Working	22 904 (65.4)	19 689 (64.8)	196 890 (64.8)
Non‐working	12 104 (34.6)	10 683 (35.2)	106 830 (35.2)
Chemotherapy^a^			
Inpatient	4744 (13.6)	3556 (11.7)	NA
Outpatient only	2584 (7.4)	2257 (7.4)	NA
None	27 546 (79.0)	24 559 (80.9)	NA
Radiation therapy^a^			
External irradiation	1261 (3.6)	946 (3.1)	NA
Brachytherapy only	11 (<0.01)	10 (<0.1)	NA
None	33 602 (96.4)	29 416 (96.9)	NA
Surgery with ≥5 days of hospitalization^a^			
Yes	12 719 (36.5)	11 170 (36.8)	NA
No	22 155 (63.5)	19 202 (63.2)	NA
Number of beds in hospital for cancer diagnosis			
<100	5376 (15.4)	4852 (16.0)	NA
≥100	29 627 (84.6)	25 515 (84.0)	NA
Unknown	5 (0.01)	5 (<0.1)	NA
Cancer diagnosis at hospital with psychiatry facilities			
Yes	18 748 (53.6)	16 187 (53.3)	NA
No	16 260 (46.4)	14 185 (46.7)	NA
First cancer diagnosis at designated cancer hospital			
Yes	17 241 (49.2)	14 870 (49.0)	NA
No	17 767 (50.8)	15 502 (51.0)	NA

*Note:* Data are n (%), unless otherwise noted.

Abbreviations: NA, not applicable; SD, standard deviation.

^a^ During the index month or the following month; percentages shown in the all‐patients cohort are based on a total of 34 874 patients with data available.

### 3.2 Time to onset of MDD

**FIGURE 2:**
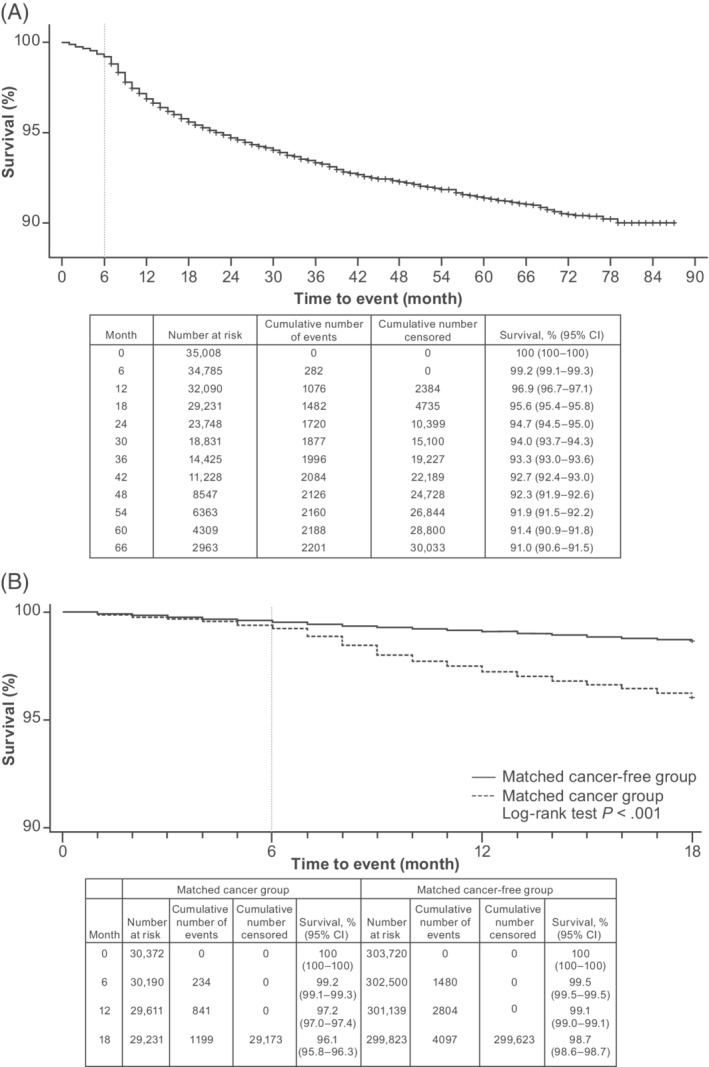
Time to MDD analysis of, A, all adult cancer patients and, B, matched cohort cancer and cancer‐free groups. The number of patients at risk, the number of MDD events, the number of patients censored, and the survival rate (ie, percentage of patients without MDD) with 95% CIs are shown for, A, every 6‐month interval during the study (data after 66 months are not shown owing to low patient numbers) and, B, every 6‐month interval during the 18‐month observation period. CI, confidence interval; MDD, major depressive disorder

The time to MDD analysis in the overall adult cancer cohort (primary endpoint) is shown in Figure 2. Among 35 008 patients with cancer, 2201 (6.3%) were diagnosed with MDD within the first 66 months (ie, up to 5 years after cancer diagnosis) of the observation period. The Kaplan‐Meier curve is shallow during the first 6 months (ie, before cancer diagnosis) but declines sharply between Month 6 and Month 18 of the observation period, corresponding to the first 12 months after cancer diagnosis (at Month 6). The number of censored patients (ie, patients who died, withdrew from health insurance, or who did not have MDD before the end of observation) increased from 4735 (14%) at Month 18 (set as end of observation period for the matched cohort) to 10,339 (30%) at Month 24.

In the matched cohort, the incidence of MDD during the 18‐month observation period was significantly greater (log‐rank test *P* < .001) in the cancer group than in the cancer‐free group (secondary endpoint; Figure 2). In the cancer group, 1199 of 30 372 (3.9%) patients were diagnosed with MDD, whereas in the cancer‐free group, 4097 of 303 720 (1.3%) patients were diagnosed with MDD. Although the largest differences in MDD incidence between the cancer and cancer‐free groups were seen after the index month, a small but significant difference was also seen in the 6 months before the index month (ie, before cancer diagnosis).

### 3.3 Multivariate analyses

#### 3.3.1 All cancers

Compared with the cancer‐free group, patients in the cancer group had a nearly threefold increased risk of being diagnosed with MDD between 6 months before and 12 months after a cancer diagnosis (HR [95% CI] 2.96 [2.77–3.16]; Table 2 and Figure 1). Both male and female cancer patients aged <40 years were more likely to be diagnosed with MDD than male cancer patients aged 40 to 64 years (Table 2). In contrast, male cancer patients aged ≥65 years were less likely to have MDD than middle‐aged male cancer patients. In all age groups, female patients were at slightly higher risk of MDD than male patients, as indicated by higher HRs (Table 2). There was no difference in the risk of MDD between working and non‐working cancer patients (Table 2).

**TABLE 2:** Multivariate analyses of time to depression within 12 months for the matched cohort

Variable	Reference	Category	Hazard ratio (95% CI)
Group	Cancer‐free group	Cancer group	2.96 (2.77–3.16)
Sex × Age	Male, 40–64	Male, <40	1.33 (1.18–1.51)
		Male, ≥65	0.65 (0.57–0.74)
		Female, <40	1.37 (1.23–1.53)
		Female, 40–64	1.11 (1.02–1.20)
		Female, ≥65	1.02 (0.87–1.20)
Working status	Working	Non‐working	1.04 (0.96–1.13)

Abbreviation: CI, confidence interval.

#### 3.3.2 Cancer type

The risk of MDD relative to cancer‐free individuals was increased in almost all cancer types (Figure 1). The highest HRs (95% CI) were seen in patients with multiple cancer types (5.89 [4.76–7.30]), pancreatic cancer (5.45 [3.70–8.04]), brain/central nervous system cancer (5.22 [3.13–8.71]), and oral cavity/pharynx cancer (4.64 [2.86–7.51]; Figure 1). The effects of sex/age and membership category in individual cancer types were generally consistent with the effects seen in all cancers combined (Table S2).

#### 3.3.3 Subgroup analysis (cancer patients only)

Within the matched cohort cancer group, the risk of MDD was significantly lower in men aged ≥65 years (HR [95% CI] 0.69 [0.53–0.90]) and lower, but not significantly, in women aged ≥65 years (0.79 [0.56–1.12]) compared with middle‐aged men, consistent with the analysis of the whole matched cohort (Table S3). The risk of MDD was significantly higher in non‐working patients than in working patients (1.33 [1.12–1.58]) and significantly lower in patients who received chemotherapy on an outpatient basis than in those who received chemotherapy on an inpatient basis (0.66 [0.52–0.83]). Surgery requiring ≥5 days of hospitalization or irradiation had no effect on the risk of MDD.

## 4 DISCUSSION

This large matched cohort study demonstrated that, compared with cancer‐free individuals, Japanese patients with cancer had an almost threefold higher risk of developing clinically significant MDD within 12 months of their cancer diagnosis. This is the first study to determine HRs for the risk of MDD in a large cohort of cancer patients in Japan. The large sample size allowed estimation of the risk of MDD in a broad range of cancer types, many of which have not been examined previously, and examination of the effects of age and sex on risk. These results underscore the need for physicians who treat cancer patients to be aware of the high possibility of MDD, particularly because MDD among cancer patients is treatable.^21^

Consistent with other studies in other populations and for specific cancer types, Japanese patients are at high risk of developing MDD after a cancer diagnosis, despite the lower prevalence of MDD in Japan generally.^15^ A Swedish matched cohort study of cancer patients diagnosed between 2001 and 2009 reported a 6.7‐fold increased risk of mental disorders, including depression, 1 week after diagnosis and a 2.2‐fold increase 1 year after diagnosis.^11^ The HR for depression at 1 year was nearly 3.0, similar to the HR in our study.^11^ Notably, the median age of patients in the Swedish study (69 years) was more than 10 years older than in our study (53 years for the matched groups). This difference reflects the employer‐based health insurance database used in the present study. Both studies, as well as other studies of specific cancer types, suggest that younger cancer patients are at higher risk of depression than older patients.^11^, ^22^, ^23^, ^24^ The risk of MDD was also somewhat higher in women than in men in our study, consistent with the Swedish study.^11^

The risk of MDD was elevated across a range of cancer types, with the highest risk seen in patients with multiple cancer sites, pancreatic cancer, brain/central nervous system cancer, and oral cavity/pharynx cancer. Understandably, the cancers with the highest risk of MDD were those with poor prognoses and/or substantial physical dysfunction. In addition, most patients with cancer at multiple sites had metastatic cancer, which is usually more advanced and associated with worse outcomes. Cancers with better prognoses (eg, thyroid cancer) generally had lower risks of MDD, although patients with breast cancer, which has a 5‐year relative survival rate^25^ and conditional 5‐year relative survival rate^26^ of 90% or more, still had a threefold higher risk of MDD compared with matched controls. Although information on cancer treatment was not analyzed in this study, it is possible that the type of therapy used to treat cancers at different sites (eg, surgery vs chemotherapy) may affect the development of MDD during the months after a cancer diagnosis. Indeed, subgroup analysis of cancer patients suggested that those who received inpatient chemotherapy within 2 months of diagnosis, reflecting more serious cancer at diagnosis, had a higher risk of MDD. Previous matched cohort studies have reported increased risk of depression in patients with uterine,^22^ head and neck,^27^ lung,^24^ colorectal,^28^, ^29^ thyroid,^30^ gastric,^31^ cervical,^32^ and breast cancer,^33^ as well as Hodgkin lymphoma.^34^ The size of our study allowed us to determine the risk of MDD for many more types of cancer than previously studied. However, the relatively low numbers of patients with some rarer types of cancer made it difficult to accurately determine HRs.

The main advantage of this study was the use of a large nationwide health insurance database, in which members could be followed across different medical facilities and irrespective of whether they visited a hospital, which allowed for a robust cohort design. The large sample size allowed analysis by cancer site, as well as multivariate analyses of the effects of age, sex, and insurance category. The high matching ratio (10:1) of cancer‐free controls to cancer patients and the random selection of controls helped minimize any potential bias. The study also included relatively long observation periods for both the overall cancer cohort (median of 32 months) and the matched cohort (18 months).

### 4.1 Study limitations

One limitation of the study is that detailed clinical data (eg, cancer stage at diagnosis, results of examination, decision by doctors, post‐diagnostic treatment, and physical function, including performance status) were not examined. Further, the method of MDD diagnosis (eg, whether a formal diagnostic interview was used) may have varied between patients. In addition, because the JMDC database primarily includes workers (and their dependents) from medium to large companies, the socioeconomic status of individuals in the study may be better than the general population in Japan. Notably, the number of older cancer patients and cancer‐free individuals was relatively small owing to the employee‐based nature of the database.

Importantly, because this study relied on a clinical diagnosis of MDD and only patients with major depression or depressive episodes were included, it provides a conservative estimate of the number of patients with depression in the study cohort. Depression can often go unrecognized, particularly if patients believe their depressive symptoms are caused by physical illness.^35^ In addition, many cancer patients may have mild depressive symptoms that are not formally diagnosed. Finally, because the study excluded patients with observation periods shorter than 18 months, it would not have included any patients who died from their cancer or from suicide or who left employment because of their physical, mental, and social condition within a year of cancer diagnosis. Therefore, the true risk of MDD in the study cohort may be higher than estimated and the time to MDD shorter.

### 4.2 Clinical implications

Compared with patients in Western countries, patients with depression in Japan may be more reluctant to seek medical treatment.^16^ As treatment of the cancer may be the immediate priority, doctors should watch for depressive symptoms in their cancer patients, especially for certain cancer types and in those receiving chemotherapy, and, when necessary, encourage patients with cancer to seek support and treatment for their depression.

## 5 CONCLUSION

In conclusion, this study documented that predominantly working‐age Japanese patients with cancer are at high risk of developing MDD in the year following a cancer diagnosis. Physicians should watch for MDD in cancer patients for at least 1 year after cancer diagnosis, when the risk of MDD is high, and treat when necessary.

## CONFLICT OF INTEREST STATEMENT

T.A. has received lecture fees from Astellas, AstraZeneca, Daiichi‐Sankyo, Dainippon‐Sumitomo, Eisai, Hisamitsu, Kyowa‐hakko Kirin, Kyowa, Eli Lilly, MSD, Meiji‐seika Pharma, Mochida, Mundipharma, Otsuka, Pfizer, Shionogi, Terumo, and Tsumura, and has received research funds from Daiichi‐Sankyo, Eisai, FUJIFILM RI Pharma, Eli Lilly, MSD, Novartis, Otsuka, Shionogi, and Tanabe‐Mitsubishi. I.M., S.F., and K.M. are employees of Takeda Pharmaceutical Company Limited.

## Supporting information


**SUPPLEMENTAL FIGURE S1** Flow diagram of cancer patients and cancer‐free controls included in the matched cohort analysis

**TABLE S1**ICD‐10 codes used to classify cancer sites

**TABLE S2**Multivariate analyses of time to depression within 12 months by cancer site for the matched cohort cancer and cancer‐free groups

**TABLE S3**Multivariate analyses of time to depression within 12 months for the matched cohort cancer group

## Data Availability

The data that support the findings of this study are available from JMDC Inc. but were used under license for the current study; therefore, restrictions apply and the data are not publicly available. For inquiries about access to the data set used in this study, please contact JMDC (https://www.jmdc.co.jp).
