# Supplementary material for: Risk of major depressive disorder in Japanese cancer patients: A matched cohort study using employer‐based health insurance claims data
Source: Psychooncology. 2020 Sep 1;29(10):1686–94. doi: 10.1002/pon.5509 (PMC7589376; doi:10.1002/pon.5509)
Supplement: Supplementary file 1 — SUPPLEMENTAL FIGURE S1 Flow diagram of cancer patients and cancer‐free controls included in the matched cohort analysis [file PON-29-1686-s001.DOCX]

**SupplementaL Figure 1** Flow diagram of cancer patients and cancer-free controls included in the matched cohort analysis

**
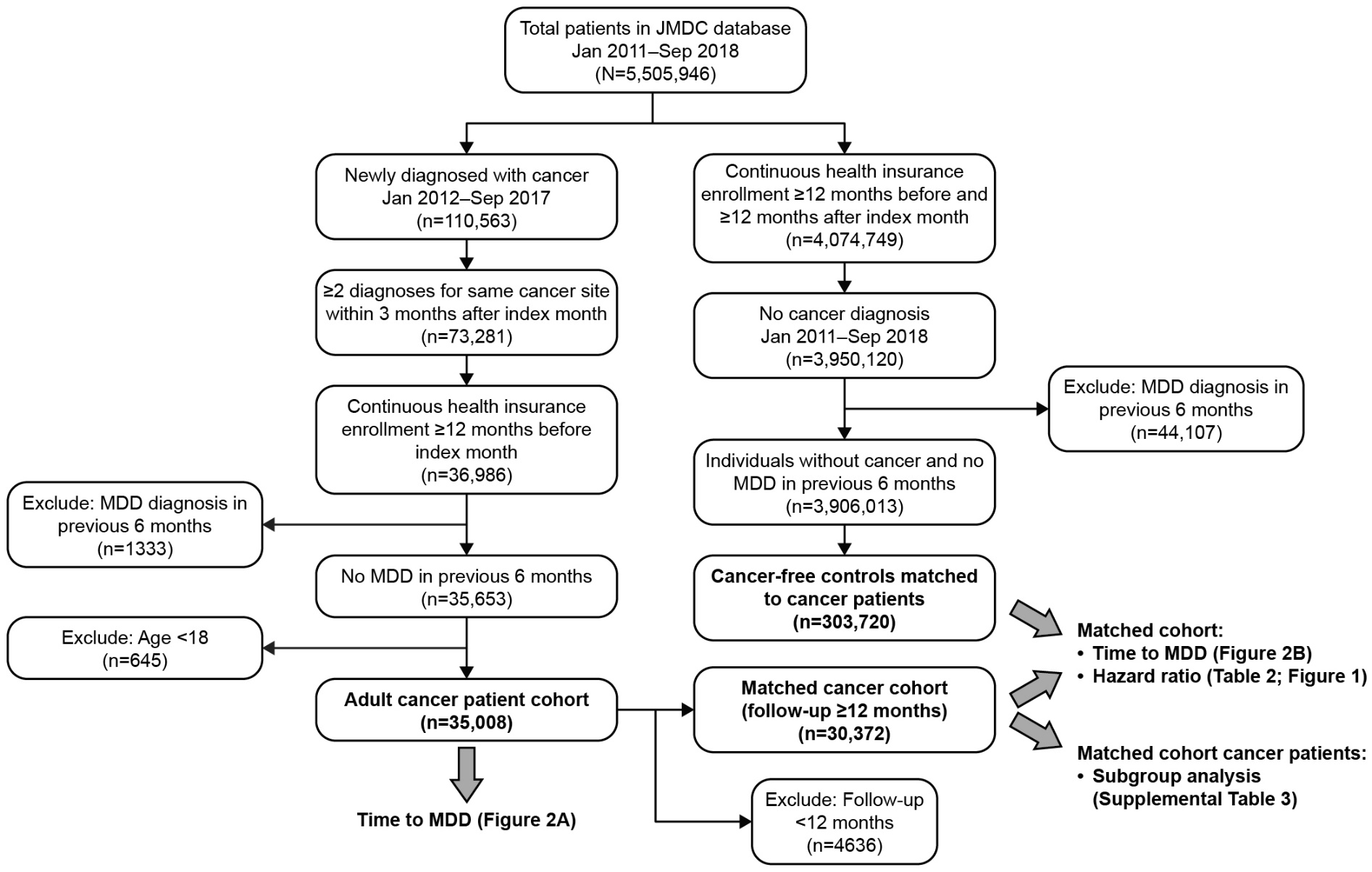
**
