# Supplementary material for: Risk of major depressive disorder in Japanese cancer patients: A matched cohort study using employer‐based health insurance claims data
Source: Psychooncology. 2020 Sep 1;29(10):1686–94. doi: 10.1002/pon.5509 (PMC7589376; doi:10.1002/pon.5509)
Supplement: Supplementary file 2 — TABLE S1ICD‐10 codes used to classify cancer sites [file PON-29-1686-s002.DOCX]

**Table S1** ICD-10 codes used to classify cancer sites

| **Site** | | **ICD-10 code** | **ICD-10 term** |  |
| --- | --- | --- | --- | --- |
| Lip, oral cavity,  and pharynx | | C00 | Malignant neoplasm of lip |  |
|  |  | C01 | Malignant neoplasm of base of tongue |  |
|  |  | C02 | Malignant neoplasm of other and unspecified parts of tongue |  |
|  |  | C03 | Malignant neoplasm of gum |  |
|  |  | C04 | Malignant neoplasm of floor of mouth |  |
|  |  | C05 | Malignant neoplasm of palate |  |
|  |  | C06 | Malignant neoplasm of other and unspecified parts of mouth |  |
|  |  | C07 | Malignant neoplasm of parotid gland |  |
|  |  | C08 | Malignant neoplasm of other and unspecified major salivary glands |  |
|  |  | C09 | Malignant neoplasm of tonsil |  |
|  |  | C10 | Malignant neoplasm of oropharynx |  |
|  |  | C11 | Malignant neoplasm of nasopharynx |  |
|  |  | C12 | Malignant neoplasm of piriform sinus |  |
|  |  | C13 | Malignant neoplasm of hypopharynx |  |
|  |  | C14 | Malignant neoplasm of other and ill-defined sites in the lip, oral cavity, and pharynx |  |
| Esophagus |  | C15 | Malignant neoplasm of esophagus |  |
| Stomach |  | C16 | Malignant neoplasm of stomach |  |
| Small intestine |  | C17 | Malignant neoplasm of small intestine |  |
| Colorectum |  |  | Colon or rectum |  |
|  | Colon | C18 | Malignant neoplasm of colon |  |
|  | Rectum | C19 | Malignant neoplasm of rectosigmoid junction |  |
|  |  | C20 | Malignant neoplasm of rectum |  |
|  |  | C21 | Malignant neoplasm of anus and anal canal |  |
| Liver |  | C22 | Malignant neoplasm of liver and intrahepatic bile ducts |  |
| Gallbladder/biliary tract | | C23 | Malignant neoplasm of gallbladder |  |
|  |  | C24 | Malignant neoplasm of other and unspecified parts of biliary tract |  |
| Pancreas |  | C25 | Malignant neoplasm of pancreas |  |
| Other digestive organs | | C26 | Malignant neoplasm of other and ill-defined digestive organs |  |
| Nasal cavity/sinus and middle ear | | C30 | Malignant neoplasm of nasal cavity and middle ear |  |
|  |  | C31 | Malignant neoplasm of accessory sinuses |  |
| Larynx |  | C32 | Malignant neoplasm of larynx |  |
| Lung |  | C33 | Malignant neoplasm of trachea |  |
|  |  | C34 | Malignant neoplasm of bronchus and lung |  |
| Other intrathoracic organs | | C37 | Malignant neoplasm of thymus |  |
|  |  | C38 | Malignant neoplasm of heart, mediastinum, and pleura |  |
| Bone and articular cartilage | | C40 | Malignant neoplasm of bone and articular cartilage of limbs |  |
|  |  | C41 | Malignant neoplasm of bone and articular cartilage of other and unspecified sites |  |
| Skin |  | C43 | Malignant melanoma of skin |  |
|  |  | C44 | Other malignant neoplasms of skin |  |
| Mesothelium and soft tissue | | C45 | Mesothelioma |  |
|  |  | C46 | Kaposi's sarcoma |  |
|  |  | C47 | Malignant neoplasm of peripheral nerves and autonomic nervous system |  |
|  |  | C48 | Malignant neoplasm of retroperitoneum and peritoneum |  |
|  |  | C49 | Malignant neoplasm of other connective and soft tissue |  |
| Breast |  | C50 | Malignant neoplasm of breast |  |
| Uterine cervix | | C53 | Malignant neoplasm of cervix uteri |  |
| Uterine corpus | | C54 | Malignant neoplasm of corpus uteri |  |
| Ovary |  | C56 | Malignant neoplasm of ovary |  |
| Other female genitalia | | C51 | Malignant neoplasm of vulva |  |
|  |  | C52 | Malignant neoplasm of vagina |  |
|  |  | C57 | Malignant neoplasm of other and unspecified female genital organs |  |
|  |  | C58 | Malignant neoplasm of placenta |  |
| Prostate |  | C61 | Malignant neoplasm of prostate |  |
| Other male genitalia | | C60 | Malignant neoplasm of penis |  |
|  |  | C62 | Malignant neoplasm of testis |  |
|  |  | C63 | Malignant neoplasm of other and unspecified male genital organs |  |
| Bladder |  | C67 | Malignant neoplasm of bladder |  |
| Kidney/urinary tract  (excl. bladder) | | C64 | Malignant neoplasm of kidney, except renal pelvis |  |
|  |  | C65 | Malignant neoplasm of renal pelvis |  |
|  |  | C66 | Malignant neoplasm of ureter |  |
|  |  | C68 | Malignant neoplasm of other and unspecified urinary organs |  |
| Eye |  | C69 | Malignant neoplasm of eye and adnexa |  |
| Brain/central nervous system | | C70 | Malignant neoplasm of meninges |  |
|  |  | C71 | Malignant neoplasm of brain |  |
|  |  | C72 | Malignant neoplasm of spinal cord, cranial nerves, and other parts of central nervous system |  |
| Thyroid gland | | C73 | Malignant neoplasm of thyroid gland |  |
| Other endocrine glands | | C74 | Malignant neoplasm of adrenal gland |  |
|  |  | C75 | Malignant neoplasm of other endocrine glands and related structures |  |
| Other malignant neoplasms | | C76 | Malignant neoplasm of other and ill-defined sites |  |
|  |  | C77 | Secondary and unspecified malignant neoplasm of lymph nodes |  |
|  |  | C78 | Secondary malignant neoplasm of respiratory and digestive organs |  |
|  |  | C79 | Secondary malignant neoplasm of other and unspecified sites |  |
|  |  | C80 | Malignant neoplasm, without specification of site |  |
| Malignant lymphoma | | C81 | Hodgkin lymphoma |  |
|  |  | C82 | Follicular lymphoma |  |
|  |  | C83 | Non-follicular lymphoma |  |
|  |  | C84 | Mature T/NK-cell lymphomas |  |
|  |  | C85 | Other and unspecified types of non-Hodgkin lymphoma |  |
|  |  | C86 | Other specified types of T/NK-cell lymphoma |  |
|  |  | C96 | Other and unspecified malignant neoplasms of lymphoid, hematopoietic, and related tissue |  |
| Multiple myeloma | | C88 | Malignant immunoproliferative diseases |  |
|  |  | C90 | Multiple myeloma and malignant plasma cell neoplasms |  |
| Leukemia |  | C91 | Lymphoid leukemia |  |
|  |  | C92 | Myeloid leukemia |  |
|  |  | C93 | Monocytic leukemia |  |
|  |  | C94 | Other leukemias of specified cell type |  |
|  |  | C95 | Leukemia of unspecified cell type |  |
| Multiple categories | | ≥2 of the above categories | | |

ICD-10, International Statistical Classification of Diseases and Related Health Problems, 10th revision.^20^
