# Supplementary material for: Risk of major depressive disorder in Japanese cancer patients: A matched cohort study using employer‐based health insurance claims data
Source: Psychooncology. 2020 Sep 1;29(10):1686–94. doi: 10.1002/pon.5509 (PMC7589376; doi:10.1002/pon.5509)
Supplement: Supplementary file 3 — TABLE S2Multivariate analyses of time to depression within 12 months by cancer site for the matched cohort cancer and cancer‐free groups [file PON-29-1686-s003.DOCX]

**Table S2** Multivariate analyses of time to depression within 12 months by cancer site for the matched cohort cancer and cancer-free groups

| **Cancer site^†^**  **Variable** | **Reference** | **Category** | **Hazard ratio**  **(95% CI)** |
| --- | --- | --- | --- |
| *Oral cavity, pharynx* | | | |
| Sex*Age | Male, 40–64 | Male, <40 | 0.94 (0.40–2.21) |
|  |  | Male, ≥65 | 0.42 (0.13–1.36) |
|  |  | Female, <40 | 0.86 (0.25–2.93) |
|  |  | Female, 40–64 | 1.00 (0.41–2.44) |
|  |  | Female, ≥65 | 1.83 (0.46–7.21) |
| Worker or dependent | Worker | Dependent | 1.05 (0.45–2.47) |
| *Esophagus* |  |  |  |
| Sex*Age | Male, 40–64 | Male, <40 | NE |
|  |  | Male, ≥65 | 0.23 (0.07–0.75) |
|  |  | Female, <40 | 5.47 (0.50–60.03) |
|  |  | Female, 40–64 | 1.65 (0.55–4.97) |
|  |  | Female, ≥65 | 1.68 (0.24–11.88) |
| Worker or dependent | Worker | Dependent | 0.52 (0.13–2.00) |
| *Stomach* |  |  |  |
| Sex*Age | Male, 40–64 | Male, <40 | 1.24 (0.74–2.09) |
|  |  | Male, ≥65 | 0.83 (0.61–1.12) |
|  |  | Female, <40 | 1.26 (0.62–2.59) |
|  |  | Female, 40–64 | 1.01 (0.70–1.46) |
|  |  | Female, ≥65 | 1.26 (0.72–2.19) |
| Worker or dependent | Worker | Dependent | 1.04 (0.70–1.54) |
| *Small intestine* | | | |
| Sex*Age | Male, 40–64 | Male, <40 | 2.10 (0.25–17.42) |
|  |  | Male, ≥65 | NE |
|  |  | Female, <40 | NE |
|  |  | Female, 40–64 | NE |
|  |  | Female, ≥65 | NE |
| Worker or dependent | Worker | Dependent | NE |
| *Colorectum* |  |  |  |
| Sex*Age | Male, 40–64 | Male, <40 | 1.21 (0.86–1.71) |
|  |  | Male, ≥65 | 0.45 (0.31–0.66) |
|  |  | Female, <40 | 1.30 (0.85–1.98) |
|  |  | Female, 40–64 | 1.02 (0.79–1.31) |
|  |  | Female, ≥65 | 0.89 (0.59–1.35) |
| Worker or dependent | Worker | Dependent | 1.07 (0.82–1.39) |
| *Liver* |  |  |  |
| Sex*Age | Male, 40–64 | Male, <40 | 0.72 (0.17–2.95) |
|  |  | Male, ≥65 | 0.77 (0.35–1.72) |
|  |  | Female, <40 | 1.91 (0.59–6.20) |
|  |  | Female, 40–64 | 0.98 (0.42–2.28) |
|  |  | Female, ≥65 | 1.12 (0.34–3.66) |
| Worker or dependent | Worker | Dependent | 0.88 (0.37–2.07) |
| *Gallbladder, bile duct* | | | |
| Sex*Age | Male, 40–64 | Male, <40 | 3.37 (0.99–11.44) |
|  |  | Male, ≥65 | 0.90 (0.30–2.65) |
|  |  | Female, <40 | 6.52 (1.76–24.07) |
|  |  | Female, 40–64 | 2.17 (0.83–5.63) |
|  |  | Female, ≥65 | 7.43 (1.93–28.57) |
| Worker or dependent | Worker | Dependent | 0.27 (0.09–0.77) |
| *Pancreas* |  |  |  |
| Sex*Age | Male, 40–64 | Male, <40 | 0.96 (0.38–2.39) |
|  |  | Male, ≥65 | 0.87 (0.46–1.67) |
|  |  | Female, <40 | 0.28 (0.04–2.13) |
|  |  | Female, 40–64 | 0.96 (0.53–1.75) |
|  |  | Female, ≥65 | 0.61 (0.22–1.66) |
| Worker or dependent | Worker | Dependent | 1.10 (0.59–2.05) |
| *Larynx* |  |  |  |
| Sex*Age | Male, 40–64 | Male, <40 | 1.74 (0.22–13.76) |
|  |  | Male, ≥65 | 0.86 (0.18–4.00) |
|  |  | Female, <40 | NE |
|  |  | Female, 40–64 | 1.11 (0.06–21.99) |
|  |  | Female, ≥65 | NE |
| Worker or dependent | Worker | Dependent | 1.19 (0.06–23.06) |
| *Lung* |  |  |  |
| Sex*Age | Male, 40–64 | Male, <40 | 1.85 (1.12–3.05) |
|  |  | Male, ≥65 | 0.58 (0.37–0.90) |
|  |  | Female, <40 | 0.91 (0.42–1.96) |
|  |  | Female, 40–64 | 0.87 (0.58–1.31) |
|  |  | Female, ≥65 | 1.10 (0.62–1.94) |
| Worker or dependent | Worker | Dependent | 1.65 (1.09–2.49) |
| *Other intrathoracic organ* | | | |
| Sex*Age | Male, 40–64 | Male, <40 | 0.57 (0.13–2.58) |
|  |  | Male, ≥65 | NE |
|  |  | Female, <40 | 0.67 (0.11–4.16) |
|  |  | Female, 40–64 | 0.24 (0.03–1.74) |
|  |  | Female, ≥65 | NE |
| Worker or dependent | Worker | Dependent | 2.36 (0.38–14.77) |
| *Skin* |  |  |  |
| Sex*Age | Male, 40–64 | Male, <40 | 0.91 (0.40–2.05) |
|  |  | Male, ≥65 | 0.54 (0.21–1.38) |
|  |  | Female, <40 | 0.95 (0.40–2.28) |
|  |  | Female, 40–64 | 1.48 (0.80–2.74) |
|  |  | Female, ≥65 | 1.49 (0.62–3.61) |
| Worker or dependent | Worker | Dependent | 1.08 (0.61–1.91) |
| *Mesothelium and soft tissue* | | | |
| Sex*Age | Male, 40–64 | Male, <40 | 1.34 (0.54–3.32) |
|  |  | Male, ≥65 | NE |
|  |  | Female, <40 | 1.68 (0.57–4.90) |
|  |  | Female, 40–64 | 2.03 (0.82–5.04) |
|  |  | Female, ≥65 | NE |
| Worker or dependent | Worker | Dependent | 0.58 (0.23–1.46) |
| *Breast* |  |  |  |
| Sex*Age | Female, 40–64 | Male, <40 | 6.21 (0.87–44.18) |
|  |  | Male, 40–64 | 0.81 (0.26–2.54) |
|  |  | Male, ≥65 | NE |
|  |  | Female, <40 | 1.31 (1.08–1.58) |
|  |  | Female, ≥65 | 0.82 (0.59–1.13) |
| Worker or dependent | Worker | Dependent | 0.96 (0.84–1.10) |
| *Uterine cervix* | | | |
| Sex*Age | Female, 40–64 | Female, <40 | 0.94 (0.68–1.31) |
|  |  | Female, ≥65 | 0.20 (0.03–1.44) |
| Worker or dependent | Worker | Dependent | 0.92 (0.67–1.27) |
| *Uterine corpus* | | | |
| Sex*Age | Female, 40–64 | Female, <40 | 1.26 (0.75–2.10) |
|  |  | Female, ≥65 | 1.18 (0.58–2.41) |
| Worker or dependent | Worker | Dependent | 1.48 (1.01–2.17) |
| *Ovary* |  |  |  |
| Sex*Age | Female, 40–64 | Female, <40 | 1.04 (0.71–1.53) |
|  |  | Female, ≥65 | 0.62 (0.19–1.96) |
| Worker or dependent | Worker | Dependent | 0.84 (0.59–1.18) |
| *Prostate gland* | | | |
| Sex*Age | Male, 40–64 | Male, <40 | 2.04 (0.29–14.60) |
|  |  | Male, ≥65 | 1.01 (0.76–1.35) |
| Worker or dependent | Worker | Dependent | 0.25 (0.04–1.79) |
| *Other male genitalia* | | | |
| Sex*Age | Male, 40–64 | Male, <40 | 1.08 (0.63–1.84) |
|  |  | Male, ≥65 | NE |
| Worker or dependent | Worker | Dependent | NE |
| *Bladder* |  |  |  |
| Sex*Age | Male, 40–64 | Male, <40 | 0.67 (0.21– 2.13) |
|  |  | Male, ≥65 | 1.00 (0.59–1.70) |
|  |  | Female, <40 | 0.82 (0.11–6.25) |
|  |  | Female, 40–64 | 2.04 (1.08–3.85) |
|  |  | Female, ≥65 | 0.90 (0.19–4.29) |
| Worker or dependent | Worker | Dependent | 0.67 (0.32–1.41) |
| *Kidney, urinary tract (except bladder)* | | | |
| Sex*Age | Male, 40–64 | Male, <40 | 0.91 (0.40–2.09) |
|  |  | Male, ≥65 | 0.50 (0.20–1.23) |
|  |  | Female, <40 | 0.83 (0.19–3.59) |
|  |  | Female, 40–64 | 1.26 (0.67–2.38) |
|  |  | Female, ≥65 | 0.41 (0.09–1.92) |
| Worker or dependent | Worker | Dependent | 1.28 (0.65–2.54) |
| *Brain, central nervous system* | | | |
| Sex*Age | Male, 40–64 | Male, <40 | 1.01 (0.53–1.94) |
|  |  | Male, ≥65 | NE |
|  |  | Female, <40 | 1.34 (0.56–3.16) |
|  |  | Female, 40–64 | 0.99 (0.39–2.53) |
|  |  | Female, ≥65 | 2.68 (0.52–13.87) |
| Worker or dependent | Worker | Dependent | 0.89 (0.40–1.97) |
| *Thyroid gland* |  |  |  |
| Sex*Age | Male, 40–64 | Male, <40 | 0.99 (0.59–1.67) |
|  |  | Male, ≥65 | 0.36 (0.05–2.60) |
|  |  | Female, <40 | 1.05 (0.68–1.61) |
|  |  | Female, 40–64 | 0.93 (0.63–1.38) |
|  |  | Female, ≥65 | 0.88 (0.39–1.96) |
| Worker or dependent | Worker | Dependent | 1.06 (0.76–1.48) |
| *Other malignant neoplasm* | | | |
| Sex*Age | Male, 40–64 | Male, <40 | 1.00 (0.46–2.16) |
|  |  | Male, ≥65 | 0.65 (0.20–2.11) |
|  |  | Female, <40 | 1.81 (0.93–3.53) |
|  |  | Female, 40–64 | 0.58 (0.29–1.17) |
|  |  | Female, ≥65 | 0.92 (0.31–2.76) |
| Worker or dependent | Worker | Dependent | 1.16 (0.63–2.13) |
| *Malignant lymphoma* | | | |
| Sex*Age | Male, 40–64 | Male, <40 | 1.87 (1.30–2.71) |
|  |  | Male, ≥65 | 0.32 (0.10–1.03) |
|  |  | Female, <40 | 1.53 (0.92–2.54) |
|  |  | Female, 40–64 | 1.14 (0.74–1.76) |
|  |  | Female, ≥65 | 1.47 (0.70–3.09) |
| Worker or dependent | Worker | Dependent | 1.25 (0.86–1.83) |
| *Multiple myeloma* | | | |
| Sex*Age | Male, 40–64 | Male, <40 | 1.08 (0.38–3.06) |
|  |  | Male, ≥65 | 0.16 (0.02–1.20) |
|  |  | Female, <40 | 1.94 (0.70–5.35) |
|  |  | Female, 40–64 | 0.80 (0.35–1.86) |
|  |  | Female, ≥65 | 0.39 (0.05–3.26) |
| Worker or dependent | Worker | Dependent | 0.79 (0.34–1.86) |
| *Leukemia* |  |  |  |
| Sex*Age | Male, 40–64 | Male, <40 | 1.00 (0.65–1.54) |
|  |  | Male, ≥65 | 0.38 (0.12–1.20) |
|  |  | Female, <40 | 0.91 (0.52–1.61) |
|  |  | Female, 40–64 | 0.54 (0.30–0.98) |
|  |  | Female, ≥65 | 0.34 (0.08–1.51) |
| Worker or dependent | Worker | Dependent | 1.72 (1.03–2.88) |
| *Multiple categories* | | | |
| Sex*Age | Male, 40–64 | Male, <40 | 1.19 (0.75–1.89) |
|  |  | Male, ≥65 | 0.80 (0.52–1.21) |
|  |  | Female, <40 | 1.17 (0.72–1.89) |
|  |  | Female, 40–64 | 0.95 (0.68–1.32) |
|  |  | Female, ≥65 | 0.78 (0.42–1.46) |
| Worker or dependent | Worker | Dependent | 1.19 (0.86–1.64) |

^†^Cancer sites with >100 patients.

NE *=* not evaluable.
