# Supplementary material for: Risk of major depressive disorder in Japanese cancer patients: A matched cohort study using employer‐based health insurance claims data
Source: Psychooncology. 2020 Sep 1;29(10):1686–94. doi: 10.1002/pon.5509 (PMC7589376; doi:10.1002/pon.5509)
Supplement: Supplementary file 4 — TABLE S3Multivariate analyses of time to depression within 12 months for the matched cohort cancer group [file PON-29-1686-s004.DOCX]

**Table S3** Multivariate analyses of time to depression within 12 months for the matched cohort cancer group

| **Variable** | **Reference** | **Category** | **Hazard ratio**  **(95% CI)** |
| --- | --- | --- | --- |
| Sex*Age | Male, 40–64 | Male, <40 | 1.26 (0.96–1.64) |
|  |  | Male, ≥65 | 0.69 (0.53–0.90) |
|  |  | Female, <40 | 1.30 (1.02–1.64) |
|  |  | Female, 40–64 | 1.06 (0.88–1.27) |
|  |  | Female, ≥65 | 0.79 (0.56–1.12) |
| Worker or dependent | Worker | Dependent | 1.33 (1.12–1.58) |
| Chemotherapy | Inpatient | None | 0.53 (0.46–0.62) |
|  |  | Outpatient only | 0.66 (0.52–0.83) |
| Radiation therapy | External irradiation | None | 0.79 (0.60–1.03) |
|  |  | Brachytherapy only | NE |
| Surgery with  ≥5 days of hospitalization | Yes | No | 1.04 (0.92–1.17) |

CI = confidence interval; NE = not evaluable.
